# Fellatio by Fruit Bats Prolongs Copulation Time

**DOI:** 10.1371/journal.pone.0007595

**Published:** 2009-10-28

**Authors:** Min Tan, Gareth Jones, Guangjian Zhu, Jianping Ye, Tiyu Hong, Shanyi Zhou, Shuyi Zhang, Libiao Zhang

**Affiliations:** 1 Guangdong Entomological Institute, Guangzhou, China; 2 School of Biological Sciences, University of Bristol, Bristol, United Kingdom; 3 College of Life Sciences, Guangxi Normal University, Guilin, China; 4 School of Life Sciences, East China Normal University, Shanghai, China; University of Exeter, United Kingdom

## Abstract

Oral sex is widely used in human foreplay, but rarely documented in other animals. Fellatio has been recorded in bonobos *Pan paniscus*, but even then functions largely as play behaviour among juvenile males. The short-nosed fruit bat *Cynopterus sphinx* exhibits resource defence polygyny and one sexually active male often roosts with groups of females in tents made from leaves. Female bats often lick their mate's penis during dorsoventral copulation. The female lowers her head to lick the shaft or the base of the male's penis but does not lick the glans penis which has already penetrated the vagina. Males never withdrew their penis when it was licked by the mating partner. A positive relationship exists between the length of time that the female licked the male's penis during copulation and the duration of copulation. Furthermore, mating pairs spent significantly more time in copulation if the female licked her mate's penis than if fellatio was absent. Males also show postcopulatory genital grooming after intromission. At present, we do not know why genital licking occurs, and we present four non-mutually exclusive hypotheses that may explain the function of fellatio in *C. sphinx*.

## Introduction

Although it is widely used in human foreplay, oral sex has rarely been recorded in non-human animals. Oral sex occurs infrequently between juvenile males or between juvenile females and juvenile males as play in bonobos *Pan paniscus* [1], [2]. Hence, to date evidence for an adaptive explanation for oral sex in animals has been lacking, and the behaviour has been considered largely specific to humans, or associated with play. Here we provide evidence that oral sex by females on males (fellatio) is routine during copulation in short-nosed fruit bats *Cynopterus sphinx* (Chiroptera: Pteropodidae), and we argue that is likely to confer adaptive benefits.

Even though the bats (Chiroptera) belong to the second largest order of mammals (>1100 species [3]), little is known about their copulatory behavior because bats are nocturnal and many roosts are inaccessible to humans. At present, descriptions of copulatory behavior exists for the hammer-headed bat *Hypsignathus monstrosus* [4], the little brown bat *Myotis lucifugus* [5], the common vampire bat *Desmodus rotundus* [6], the Indian flying fox *Pteropus giganteus* [7], and the Brazilian free-tailed bat *Tadarida brasiliensis* [8], [9]. Specialized behaviors associated with reproduction, including harem-forming, tent-making, and territorial defence appear during the breeding season in some bat species [10]–[12].

The short-nosed fruit bat *C. sphinx* roosts in tents made from foliage that are constructed and defended by males to attract females as a form of resource defense polygyny [13]. Moreover, the bats transport pollen and disperse the seeds of plants [14], and hence have great value in ecosystems. *C. sphinx* is listed as ‘Near Threatened’ in China, and almost meets the criteria for being considered ‘Vulnerable’ [15]. Gopukumar & Balasingh discussed the mating strategy adopted by *C. sphinx* and the mechanics of tent-making [12]. Some recent studies found that nonharem males were actively involving in female recruitment and also obtained some reproductive success [16], [17]. Based on this information, we wanted to know more about the nature of copulation in this bat species. We observed that females were not passive during copulation but performed oral sex, licking their mate's penis during copulation. This paper will provide information on the copulatory behavior and the duration of copulations of *C. sphinx* under captive conditions, highlighting the possible functions of this unusual licking behavior.

## Materials and Methods

### Ethics Statement

The research presented in this manuscript was conducted according to protocols approved by the Guangdong Entomological Institute Administrative Panel on Laboratory Animal Care.

### Study site

We conducted this study in January 2007 and from November 2007 to December 2007 in Guangzhou City (23°08′N, 113°15′E), Guangdong Province, southern China. The city experiences a south subtropical monsoon climate. Ornamental Chinese fan-palm trees are widespread in parks and on the university campus, and these are important roosting sites for *C. sphinx* [18]–[20].

### Bat capture and marking

We captured bats from Yuexiu Park (23°08′N, 113°15′E, 70 m a.s.l., 8 km from the laboratory) during daytime, and recorded the sex, age and reproductive state of all individuals. Subadults, pregnant females and lactating females were excluded from this research to avoid limiting the opportunities for copulation. Thirty males and thirty females were marked with split metal bat rings on their left (males) or right arms (females) and were held in thirty flight cages (2.9 m long ×2.4 m wide ×2.0 m in height) made of wire, with each cage holding a male and female that were paired at random. The flight cages were exposed to natural conditions and several leaves of Chinese fan-palm were hung on the ceilings of the cage to provide roosts for the bats. We changed the leaves at regular intervals. The bats were fed with bananas and water with added vitamin supplements ad libitum every day. During the study, the average humidity was 53.7±2.0% and average temperature was 20.2±0.5C (both mean±SE, N = 32). After the investigation, bats were released at the place of capture or used for other studies.

### Behavioural observations

We observed behaviours at roosts by using a PICO2000 series multimedia digital video monitor connected to a computer. From 19.00 h to 07.00 h the next morning, we recorded behaviours continuously. In the early night, data were recorded directly by using scan sampling methods [21], with a 5 minute interval between scans. We also measured the frequency and duration of copulatory behaviours. The duration of copulation was defined as when the penis entered the vagina until when it withdrew completely. In the late night (from 24.00 h to 07.00 h), the PICO2000 series multimedia digital video monitoring system recorded the behaviours and transferred data to computers automatically for later analysis.

### Statistical analysis

Twenty successful copulations were observed among these 30 pairs, and each copulation was from a different pair. We tested all data for normality, and used parametric analyses (Pearson correlation) and linear regression to determine whether the total length of time that the female licked the male's penis during copulation and the total duration of copulation were associated. Nonparametric tests (Mann-Whitney U test) were used when data were not distributed normally. Means are given with their standard error, and all tests were conducted at the 0.05 significance level.

## Results

During copulation, the pair appeared to move forwards and backwards uninterruptedly and rhythmically. When a male was chewing or severing the Chinese fan-palm leaves to make a tent, or when males crawled upon the upper surface of a tent or were grooming themselves within a tent, a female would fly to the tent, stretch her wings, move her head slowly towards the male, and then sniff the male's face and neck. Subsequently, the pair's heads extended towards each other and the bats would lick one another. At this time, the male would make approaches to the female with his thumbs. After moving onto the female's back, the male would adjust his position to find a more appropriate copulatory posture, so that the pair would adopt a face-to-back mating posture. Sometimes the female appeared to resist, or even escaped by accident, and then the male would follow her until copulation was completed. In two instances, the female evaded the male for about eight to 25 seconds and turned to bite the male, but later the male followed her until mating was completed. In four instances, the female appeared to resist the male's approaches, but did not evade successfully, and copulations were eventually completed. During the copulation, the male usually maintained a tight hold on the scruff of the female's neck by using his mouth, and his thumbs held her wings firmly. When copulation was completed, the male licked his penis for several seconds. This self-licking occurred after all of 20 copulations, but was absent after three instances in which intromission failed to occur. Subsequently, the male often groomed himself or licked the inner surface of the tent, yet seldom flew away. Also, the female groomed herself and typically stayed close to her mate.

**Figure 1:**
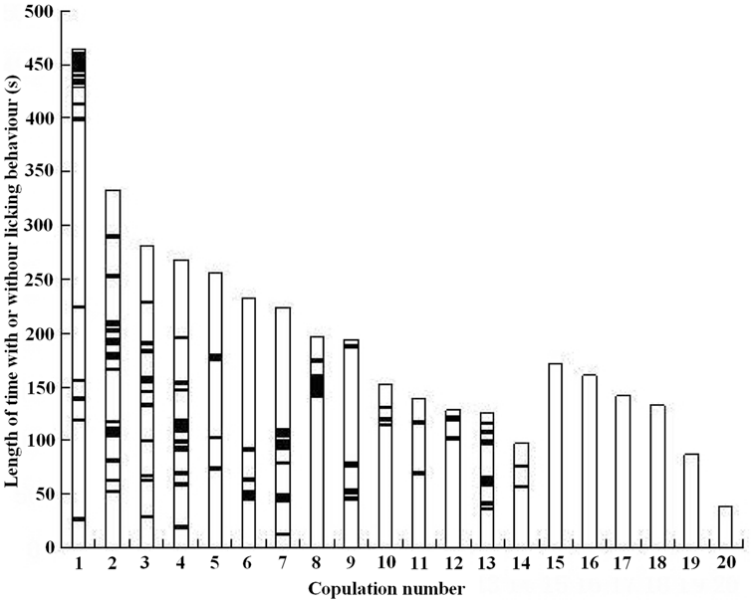
Frequency distributions of the time that female *Cynopterus sphinx* licked (black portions) and did not lick (white portions) the male's penis in 20 copulation attempts.

**Figure 2:**
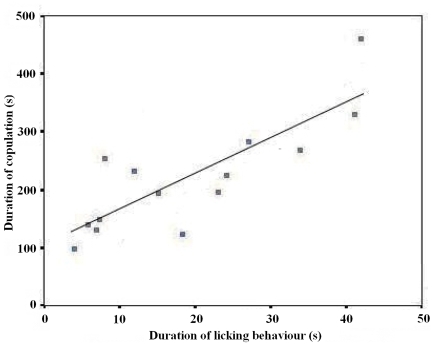
Relationship between the duration of copulation and total length of time that the female licked the male's penis in each copulation (N = 14). The relationship is described by the equation: duration of copulation (s) = 101.24+6.22 (duration of licking behaviour, (s)) (*F*_1,13_ = 26.14, P<0.001). Therefore each second of licking prolongs copulation by approximately 6 s.

**Figure 3:**
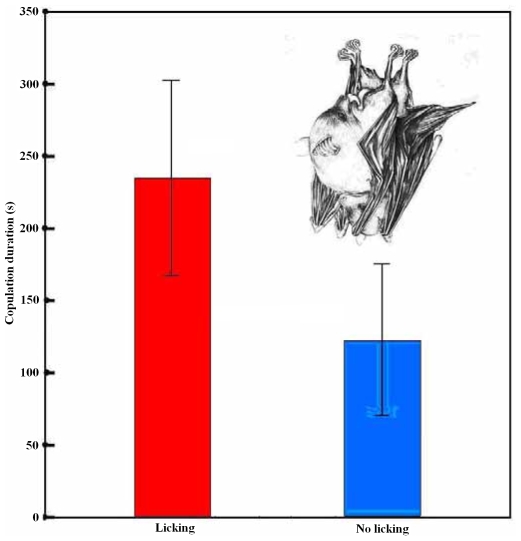
Copulation duration in *Cynopterus sphinx* according to whether the female licks the male's penis (Licking) or not (No licking). Means and standard errors are shown. Vignette shows a female performing fellatio, drawn by Mei Wang.

Interestingly, we found that the female lowered her head to lick the shaft or the base of male penis frequently during copulation (Video S1). The male never withdrew when his penis was licked by the female. Genital licking was observed in 14 of 20 copulations, whereas six copulations did not involve licking behavior (Fig. 1). The average duration of penis licking was 19.14±3.45 s, representing about 8.7% of the average duration of copulation (220.29±26.19 s (N = 14)). There was a strong correlation between the total length of time that the female licked the male's penis and the duration of copulation (Pearson correlation: r_12_ = 0.828, P<0.001: Fig. 2). Therefore the longer the female licked the penis of her mate, the longer they copulated for. Furthermore, we found that whether a female licked her mate's penis during copulation had a significant influence on the duration of copulation. The pairs spent more time copulating if the female licked her mate's penis (220.29±26.19 sc, N = 14) than on occasions when females did not show licking behavior (121.83±20.56 s, N = 6: Mann-Whitney U test: P = 0.039; Fig. 3). This result suggests that the licking behaviour may play an important role in copulation by prolonging intromission.

## Discussion

We found that female short-nosed fruit bats *C. sphinx* lick their mate's penis regularly during copulation, and that each second of licking results in approximately 6 extra seconds of copulation. Copulations also last longer if licking occurs than when no licking takes place. Our observations are the first to show regular fellatio in adult animals other than humans.

The duration of copulation in *C. sphinx* (100–300 s) is much longer than in the Indian flying fox *P. giganteus* (30–40 s), and the hammer-headed bat *H. monstrosus* (30–60 s) [4], [7]. The Indian flying fox and the hammer-headed bat were studied in the field however, and male-male competition in colonies may reduce copulation time. Bats may also shorten copulation time to reduce predation risk in the field, especially if vigilance is reduced during copulation.

Our observations showed that the female frequently lowered her head to only lick the penile shaft during intromission, because the glans penis has already entered the vagina. In six instances licking behavior was not apparent, maybe because the female was forced to copulate and sometimes evaded successfully for several seconds. There are few observations of fellatio in non-human animals. Oral sex was found as a play behaviour between juvenile males or between juvenile female and juvenile male bonobos *Pan paniscus* [1], [2]. Schürmann observed that a female orangutan, *Pongo pygmaeus*, often interrupted copulation for a short while, manipulating her mate's penis with her hand, licking it or putting it in her mouth before mounting again. It is plausible that this female's behavior increased male arousal [22]. In general, many animals may lick genitals before and after copulation, for example, the male of ring-tailed lemur, *Lemur catta*, often licks the genitals of the female in order to judge whether she is in oestrous, and after copulation, he also licks his penis [23]. Both male and female Livingstone's fruit bats (*Pteropus livingstonii*) also licked the genitals of their partners during heterosexual interactions [24], [25]. However, because of the limited details provided, we do not know whether fellatio occurs during copulation in these species. The bat penis contains erectile tissue (corpus cavernosa and corpus spongiosum) which is similar to that found in primates and humans. If the erectile tissue is stimulated during copulation e.g. by rhythmic vaginal contraction, it will increase the rigidity of the penis, and maintain the erection for longer [26]. We speculate that the female *C. sphinx* licks the male penis to increase penile stimulation, stiffening the penis and maintaining the male's erection. At the same time, the female's saliva may increase lubrication, thus facilitating intromission and thrusting. In combination, these features may prolong copulation in *C. sphinx*.

So in a similar way to the anthropoid primates [27], the *C. sphinx* females (like most animals, see [28]) are not passive during copulation but rather communicate with the male, in this case by licking his penis. We propose a series of adaptive hypotheses to explain genital licking in *C. sphinx*. First, genital licking may lubricate the penis or increase penile stimulation, prolonging the duration of copulation. Prolonged copulation might assist sperm transport from the vagina to the oviduct, or stimulate secretions of the pituitary gland in the female [26] and hence increase the likelihood of fertilization. Second, prolonged copulation might be a method of mate-guarding, because the mates would normally segregate after copulation to form unisexual groups which persist throughout the non-breeding season [29]. Third, fellatio may confer bactericidal benefits and assist in the prevention of sexually transmitted diseases (STDs) both to females [30]–[33], and to males that lick their own penis briefly after copulation [34]–[37]. Saliva has a protective repertoire that goes beyond antibacterial activity to include antifungal, antichlamydial, and antiviral properties as well [38]. Finally, genital licking may facilitate the detection and identification of MHC-dependent chemical cues associated with mate choice [39], [40].

In conclusion, we have documented fellatio in animals that may have functional significance. Of course, adaptive benefits remain unproven until tested, ideally by experimentation, but our study identifies potential avenues to explore if the null hypothesis of no benefit (e.g. via low cost to donor and recipient) is to be rejected. We believe that ours is the first large scale observational study of oral sex in non-humans, and we extend the interpretation of such behaviour beyond that of ‘pleasure giving’ into an evolutionary context. Importantly we show that fellatio increases the duration of copulation. This may have several important functions, for example increasing fertilization success, or even reducing the risk of contracting STDs. The behaviour presumably favours the donor, although it may also benefit both partners especially if fertilization success is increased. It is conceivable that the female manipulates the male by increasing sexual stimulation, so that she ultimately benefits.

## Supporting Information

Video S1 The face-to-back copulation of Cynopterus sphinx, showing the fellatio by female
(3.04 MB MPG)
